# Unexpected Cement Fragment in the Heart: A Case Report of Intracardiac Embolism

**DOI:** 10.7759/cureus.111843

**Published:** 2026-06-30

**Authors:** Mohamadanas Oudih, Mouaz Oudih, Mustafa Al-Taie, Fathima Jahufar, Snehal Patel

**Affiliations:** 1 Internal Medicine, Ball Memorial Hospital/ Indiana University, Muncie, USA; 2 Internal Medicine, Ascension Illinois Saint Joseph Hospital, Chicago, USA; 3 Cardiology, Louisiana Children's Medical Center (LCMC) Health Center/Tulane University, New Orleans, USA; 4 Cardiology, Montefiore Medical Center, Albert Einstein College of Medicine, New York, USA

**Keywords:** bone cement implantation syndrome, cardiovascular medicine, endocarditis, intracardiac cement embolism, kyphoplasty

## Abstract

Intracardiac cement embolism (ICE) is a rare complication of vertebroplasty and kyphoplasty. We present an unusual case of ICE in a 54-year-old woman following spinal surgery for osteodystrophy and osteomyelitis. Postoperative imaging revealed cement migration to the right atrium. Anticoagulation was initiated but discontinued due to bleeding. With no clear guidelines and high surgical risk, conservative management was pursued. This case highlights the importance of early recognition and the complexities of managing ICE in high-risk patients.

## Introduction

Bone cement implantation syndrome (BCIS) is a rare but potentially life-threatening complication associated with the intraoperative use of polymethylmethacrylate (PMMA), most commonly during cemented arthroplasty and vertebral augmentation procedures. Clinically, BCIS encompasses a broad spectrum of intraoperative and early postoperative manifestations, ranging from transient hypoxia and hypotension to severe cardiovascular collapse, including syncope, malignant arrhythmias, and cardiac arrest [1-3]. Its pathophysiology is multifactorial, with proposed mechanisms including monomer-mediated toxicity, histamine release, and embolic phenomena.

Intracardiac cement embolism (ICE), in contrast, is a distinct embolic complication characterized by the migration and lodgment of cement material within the cardiac chambers or great vessels. Although ICE may occur in the setting of BCIS-related cement and fat embolization, it is not synonymous with BCIS and should be regarded as a focal mechanical complication within the broader spectrum of cement-related embolic events. Among the proposed mechanisms of BCIS, the embolic theory is most directly relevant to ICE, as cement or associated debris may enter the venous circulation and subsequently migrate to the right heart or pulmonary vasculature, resulting in mechanical obstruction and local tissue injury [3].

Cement leakage during vertebral augmentation procedures, such as vertebroplasty and kyphoplasty, is relatively common, occurring in up to 65% of cases. In contrast, clinically significant cement embolization to the right heart or pulmonary circulation is much less frequent, occurring in approximately 3.9% of patients [4,5]. Once embolized, cement fragments may lodge within the cardiac chambers or pulmonary arteries or, in rare cases, perforate cardiac structures, resulting in clinical presentations that range from asymptomatic to life-threatening and may occur immediately or be delayed for years after the initial procedure [6].

This report describes a rare case of right atrial ICE complicated by interval fragmentation and subsequent suspected infective endocarditis. The case highlights the diagnostic challenges, individualized management considerations, and potential delayed complications associated with this uncommon condition.

This article was previously presented as a meeting abstract at the 2024 Heart Failure Society of America (HFSA) Annual Scientific Meeting in September 2024.

## Case presentation

A 54-year-old woman with a history of end-stage renal disease (ESRD) on peritoneal dialysis, hypertension, and hyperlipidemia presented to the emergency department with a two-week history of back pain and progressive lower extremity weakness. On arrival, she was afebrile and not hypoxic, with a blood pressure of 81/60 mmHg and a heart rate of 100 beats/min. Physical examination was unremarkable. Neurological examination was notable only for 4/5 motor weakness in the lower extremities. Initial blood cultures grew gram-positive bacilli, later identified as *Bacillus cereus*. Magnetic resonance imaging of the spine demonstrated a kyphotic deformity at T8-T10 consistent with renal osteodystrophy and osteomyelitis.

After medical stabilization, the patient underwent T8-T10 laminectomy, T9-T10 decompression, and T5-L1 posterior internal fixation with vertebral cement augmentation. During cement injection, retrograde flow into a segmental vein was observed, and cement delivery was immediately discontinued upon visualization of venous opacification.

Three days postoperatively, although the patient remained asymptomatic, follow-up contrast-enhanced CT of the thoracic spine demonstrated a linear hyperattenuating cement fragment extending cranially from the paravertebral venous system into the right atrium, confirming venous embolization without evidence of pericardial effusion or cardiac perforation (Figure 1). Transthoracic echocardiography demonstrated a well-circumscribed, non-mobile 4.1 cm linear echogenic structure projecting toward the tricuspid valve region without definite evidence of valvular involvement or cardiac perforation (Figure 2). The combination of documented intraoperative venous cement leakage, the characteristic hyperattenuating linear structure extending along the venous system into the right atrium on CT, and the corresponding echocardiographic findings established the diagnosis of ICE.

**Figure 1:**
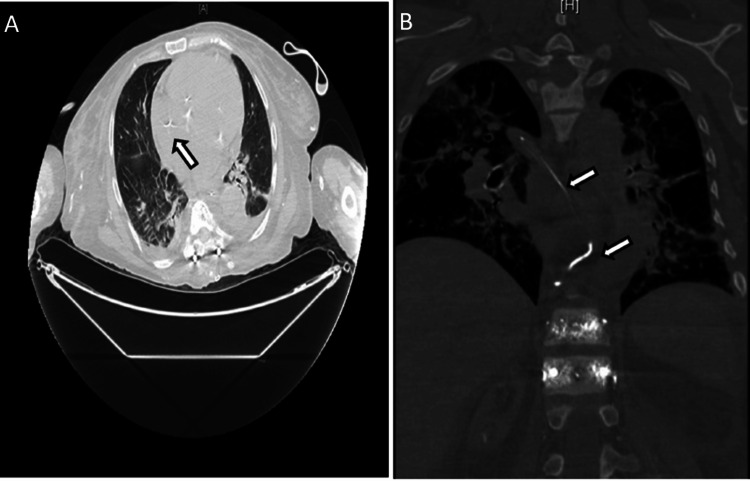
(A, B) Contrast-enhanced axial and coronal CT images of the chest and thoracic spine demonstrating intracardiac cement embolism. Hyperattenuating polymethylmethacrylate (PMMA) cement is seen extending from the paravertebral venous plexus through the systemic venous circulation into the right atrium (arrows), confirming venous cement embolization. Extraosseous cement extension into the epidural space is also present. No pericardial effusion or CT evidence of cardiac perforation is identified.

**Figure 2:**
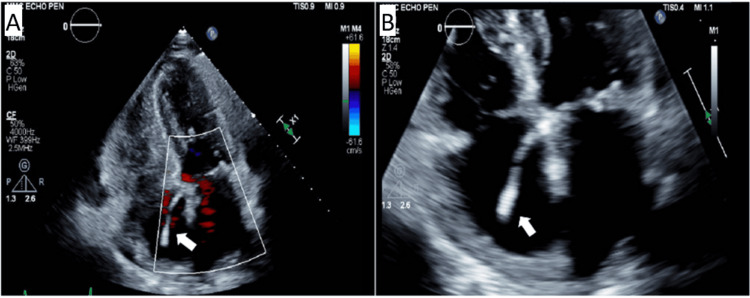
(A, B) Transthoracic echocardiogram (apical four-chamber view) demonstrating intracardiac cement embolism. Arrows identify a well-circumscribed, non-mobile, linear echogenic structure measuring 4.1 × 0.7 cm within the right atrium, projecting toward the tricuspid valve region without definite echocardiographic evidence of valvular involvement or cardiac perforation.

Given the absence of established management guidelines, the cardiology team recommended anticoagulation with a continuous heparin infusion to reduce the risk of secondary thrombus formation on the cement fragment. The infusion was discontinued because of a progressive decline in hemoglobin. Follow-up cardiac CT demonstrated interval fragmentation of the embolus, with a curvilinear cement fragment remaining within the right atrium and the fractured distal segment terminating near the junction of the interatrial and interventricular septum. There was no evidence of extension beyond the cardiac silhouette or associated pericardial effusion (Figure 3).

**Figure 3:**
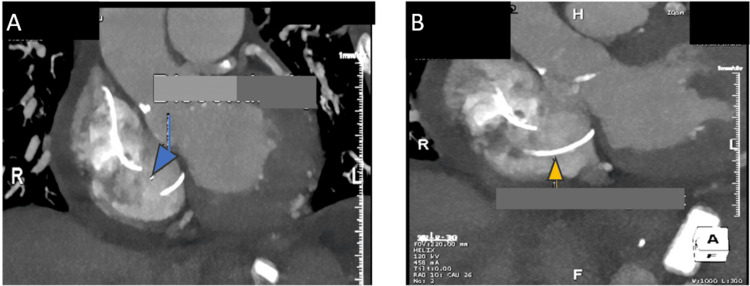
(A, B) Contrast-enhanced cardiac CT demonstrating interval fragmentation of the intracardiac cement embolus. The blue arrow identifies the residual curvilinear hyperattenuating cement fragment within the right atrium. The yellow arrow indicates the fractured distal segment (approximately 3.1 cm) terminating near the junction of the interatrial and interventricular septum. No CT evidence of extension beyond the cardiac silhouette or associated pericardial effusion is present.

Cardiothoracic surgery and interventional radiology were consulted to evaluate both surgical and percutaneous treatment options. After multidisciplinary evaluation, both surgical and endovascular retrieval were considered high risk because of interval embolus fragmentation, persistent anemia, and the patient's extensive comorbidities. Conservative management was therefore recommended, and the patient was discharged four weeks later in stable condition.

One month after discharge, the patient was readmitted with altered mental status and septic shock. Repeat transthoracic echocardiography demonstrated a new 2.7 cm linear mass attached to the coronary sinus that was suspicious for vegetation. The patient was treated with intravenous antibiotics for suspected infective endocarditis but subsequently developed refractory septic shock and died.

## Discussion

ICE is a rare but potentially life-threatening complication of vertebroplasty and kyphoplasty, occurring in approximately 3.9% of patients undergoing these procedures [4,5]. It results from PMMA leakage into the vertebral venous plexus, allowing cement to migrate through the systemic venous circulation to the right heart or pulmonary vasculature [6]. Although some patients remain asymptomatic, ICE can result in serious complications including arrhythmias, myocardial infarction, stroke, cardiac perforation, valvular injury, pulmonary embolism, and death [1,3].

Our case illustrates several challenges associated with ICE. Despite prompt cessation of cement injection after venous opacification was recognized intraoperatively, the patient developed a large (4.1 cm) right atrial cement embolus confirmed by CT and echocardiography. The differential diagnosis of a linear echogenic right atrial structure includes thrombus, infective vegetation, retained catheter fragments, calcification, and intracardiac foreign bodies. In our patient, the combination of documented intraoperative venous cement leakage, the characteristic hyperattenuating linear structure extending from the paravertebral venous system into the right atrium on CT, and the corresponding echocardiographic findings strongly supported the diagnosis of ICE while excluding these alternative etiologies. Although transthoracic echocardiography was valuable for detecting the intracardiac lesion and assessing its size, location, and mobility, cardiac CT provided superior anatomical characterization by demonstrating the characteristic high-density cement fragment and its subsequent fragmentation, thereby confirming the diagnosis and guiding management.

Similar to previously reported cases, our patient remained asymptomatic immediately after the procedure, highlighting the variable and often delayed clinical presentation of ICE [7,8]. While some patients develop immediate and severe manifestations following cement embolization [6,9-11], others, as in our case, remain clinically silent until delayed complications arise. This variability complicates clinical decision-making, as emboli may migrate or fragment over time, increasing the risk of subsequent complications, including infective endocarditis [12].

The management of ICE remains challenging because standardized treatment guidelines are lacking [7,8]. Instead, treatment should be individualized according to embolus location, mobility, associated complications, and the patient's overall clinical condition. In published reports, open-heart surgery has primarily been reserved for patients with cardiac perforation, tamponade, valvular injury, or large mobile emboli [13,14]. Endovascular retrieval has been successfully performed in selected patients with right-sided emboli when the fragment was intact and accessible and the procedural risk was acceptable [15,16]. Conversely, conservative management, with or without anticoagulation, has generally been adopted for asymptomatic patients, incidentally detected emboli, or those with significant comorbidities that render intervention high risk [17].

Our patient's management was consistent with the latter approach. Anticoagulation with heparin was initiated to reduce the risk of secondary thrombus formation on the cement fragment rather than to treat the cement embolus itself but was discontinued because of persistent anemia. Follow-up cardiac CT demonstrated interval fragmentation of the embolus. After multidisciplinary evaluation by cardiothoracic surgery and interventional radiology, both surgical and endovascular retrieval were considered to carry excessive procedural risk because of the fragmented embolus, the potential for further fragmentation during manipulation, persistent anemia, and the patient's significant comorbidities. Consequently, conservative management was pursued.

Although several conservatively managed cases have been reported with favorable outcomes [17], our patient was readmitted one month later with septic shock and a new linear mass adjacent to the coronary sinus suspicious for vegetation. While the intracardiac cement embolus may have served as a potential nidus for infection, this association remains presumptive given the patient's multiple competing risk factors, including ESRD, prior *B. cereus* bacteremia, osteomyelitis, and recent spinal surgery. She ultimately died from septic shock, underscoring that conservative management may be appropriate in selected patients but does not eliminate the risk of serious delayed complications.

Overall, this case highlights the importance of early recognition, multimodality imaging, and multidisciplinary decision-making in patients with ICE. In the absence of evidence-based treatment guidelines, management should be individualized by balancing embolus characteristics, procedural feasibility, patient comorbidities, and anticipated prognosis.

## Conclusions

ICE is a rare but potentially life-threatening complication of vertebral augmentation procedures that requires prompt recognition and careful multidisciplinary evaluation. Because standardized management guidelines are lacking, treatment should be individualized based on embolus location, mobility, associated complications, technical feasibility of retrieval, patient comorbidities, and overall prognosis. Available treatment strategies include conservative management, anticoagulation to reduce the risk of secondary thrombus formation, endovascular retrieval, and surgical extraction, each with specific risks and benefits. Our case highlights the complexity of clinical decision-making when multiple therapeutic options are limited by embolus fragmentation and significant patient comorbidities. It also underscores the importance of multimodality imaging for diagnosis and follow-up, as well as the potential for delayed complications despite initially conservative management. Additional studies are needed to better define evidence-based management strategies for this uncommon but clinically significant condition.
